# Mobile Phone Technologies in the Management of Ischemic Heart Disease, Heart Failure, and Hypertension: Systematic Review and Meta-Analysis

**DOI:** 10.2196/16695

**Published:** 2020-07-06

**Authors:** Praveen Indraratna, Daniel Tardo, Jennifer Yu, Kim Delbaere, Matthew Brodie, Nigel Lovell, Sze-Yuan Ooi

**Affiliations:** 1 Department of Cardiology Prince of Wales Hospital Sydney Australia; 2 Prince of Wales Clinical School The University of New South Wales Sydney Australia; 3 Department of Medicine St Vincent's Hospital Sydney Australia; 4 Faculty of Medicine The University of Notre Dame Sydney Australia; 5 Falls, Balance and Injury Research Centre Neuroscience Research Australia Sydney Australia; 6 School of Public Health and Community Medicine University of New South Wales Sydney Australia; 7 Graduate School of Biomedical Engineering The University of New South Wales Sydney Australia

**Keywords:** mobile phone, text messaging, telemedicine, myocardial ischemia, heart failure, hypertension

## Abstract

**Background:**

Cardiovascular disease (CVD) remains the leading cause of death worldwide. Mobile phones have become ubiquitous in most developed societies. Smartphone apps, telemonitoring, and clinician-driven SMS allow for novel opportunities and methods in managing chronic CVD, such as ischemic heart disease, heart failure, and hypertension, and in the conduct and support of cardiac rehabilitation.

**Objective:**

A systematic review was conducted using seven electronic databases, identifying all relevant randomized control trials (RCTs) featuring a mobile phone intervention (MPI) used in the management of chronic CVD. Outcomes assessed included mortality, hospitalizations, blood pressure (BP), and BMI.

**Methods:**

Electronic data searches were performed using seven databases from January 2000 to June 2019. Relevant articles were reviewed and analyzed. Meta-analysis was performed using standard techniques. The odds ratio (OR) was used as a summary statistic for dichotomous variables. A random effect model was used.

**Results:**

A total of 26 RCTs including 6713 patients were identified and are described in this review, and 12 RCTs were included in the meta-analysis. In patients with heart failure, MPIs were associated with a significantly lower rate of hospitalizations (244/792, 30.8% vs 287/803, 35.7%; n=1595; OR 0.77, 95% CI 0.62 to 0.97; *P*=.03; I^2^=0%). In patients with hypertension, patients exposed to MPIs had a significantly lower systolic BP (mean difference 4.3 mm Hg; 95% CI −7.8 to −0.78 mm Hg; n=2023; *P*=.02).

**Conclusions:**

The available data suggest that MPIs may have a role as a valuable adjunct in the management of chronic CVD.

## Introduction

### Background

Cardiovascular disease (CVD) is the leading cause of death worldwide [1] and is a leading cause of hospital readmission. With an aging population and a rising prevalence of obesity in developed countries, the physical and economic burden of CVD will only increase. Repeat cardiac events contribute significantly to the burden of disease [2], and reductions of more than 80% of such events may be achieved through secondary prevention [3]. Secondary prevention relies upon monitoring and control of modifiable risk factors such as hypertension, dyslipidemia, and obesity. This is achieved through (1) patient education and empowerment, (2) the prescription of and compliance with optimal pharmacotherapy, (3) lifestyle modification, and (4) the early identification of clinical deterioration.

Traditional cardiac rehabilitation (CR) programs are the embodiment of these principles, offering support, tailored education, and supervised exercise [4]. They are proven to reduce repeat events and mortality, and their adoption is strongly advocated in the current guidelines for the management of acute coronary syndromes [5]. However, globally, CR participation rates are, at best, 30% [6]. The reasons for this are varied, but importantly in the context of this review, they include accessibility, convenience, patient availability, and a fear of group-based settings [7]. Therefore, novel strategies are required.

The outpatient management of heart failure is ideally performed using a multidisciplinary approach, including nurse-led medication titration services, which reduce readmission rates and mortality. However, these services are heavily dependent upon the limited resources of skilled nursing staff. Again, a novel approach to outpatient management is required.

The telemedicine care process involves using communication networks to deliver health care services and move patient information between locations. Literature reviews have underlined several advantages of using telemedicine to reduce inequalities in cardiovascular outcomes [8] and provide improved care for patients with heart failure [9]. Systematic review evidence has found telemedicine as a cost-effective option for treating many chronic conditions [10]. However, not all remote monitoring apps have equal efficacy, and the reported benefits depend on the type of technology used, the presence of organizational support, and the level of care provided to control groups [11].

Mobile phones (particularly smartphones) provide new opportunities to remotely care for patients with cardiac conditions. Traditionally, telemedicine required the provision of home-based specialized monitoring equipment to patients. Smartphones, mobile phones, and wearable technology, however, offer tremendous potential for monitoring health through phone calls, text messages, data recording, highly portable peripheral devices, and activity monitoring, which may find utility for novel models of health care delivery that are cost-effective, accessible, and patient-centric. Mobile phones are ubiquitous, and the recent landmark Nature publication [12] highlights the staggering potential for big data to encourage people to be physically active and to influence health policy. However, concerns exist regarding the use of and inconsistencies in unvalidated smartphone-based interventions for health research [13]. Mobile phone apps have been used to target individuals with ischemic heart disease, cardiac failure, and hypertension and in those undergoing CR; however, gaps exist between the reported potential for mobile phone technology to transform health care services and current clinical practice. The evidence for improved health outcomes now needs to be systematically assessed, and consequently, no clear guidelines exist on the use of these new technologies in clinical practice.

### Objectives

The purpose of this study was to systematically review and meta-analyze the evidence for mobile phone technology in the management of cardiac conditions according to the following questions: (1) What are the specific interventions available and do they involve an interface whereby the clinician can use the data to intervene (henceforth referred to as a *back-end*)? (2) Can mobile phone technologies improve patient outcomes with respect to mortality and hospitalizations? and (3) Can mobile phone technologies reduce risk factors for cardiac events, specifically medication compliance and hypertension?

## Methods

### Data Search Strategy

Electronic data searches were performed using Ovid MEDLINE, PubMed, EMBASE, Database of Abstracts of Review of Effects, American College of Physicians Journal Club, National Health Service Economic Evaluation Database, and Cochrane Database of Systematic Reviews from January 2000 to June 2019. A combination of search terms were used to maximize sensitivity: *mobile applications*, *cell phones*, *smart phones*, *mobile phones*, and *text messaging* were combined with *cardiovascular disease*, *heart failure*, *ischemic heart disease*, *acute coronary syndrome*, *myocardial infarction*, *cardiac rehabilitation*, and *hypertension* as either Medical Subject Headings (MeSH) terms or key words. Full texts of selected publications were retrieved following review of all abstracts. A manual review of the reference lists of each relevant manuscript was performed to identify further results. Recording of results followed the Preferred Reporting Items for Systematic Reviews and Meta-Analyses (PRISMA) statement. Bias assessments were based on the Cochrane Risk of Bias assessment tool (RoB 2).

### Selection Criteria

Studies were considered eligible for this systematic review if a mobile phone app or text messaging (used interchangeably henceforth with SMS) were used in a randomized control trial (RCT) in the management of ischemic heart disease, cardiac failure, or hypertension in adult patients. Studies limited to the management of obesity, dyslipidemia, diabetes, sedentary lifestyle, and smoking cessation in patients without CVD were not included. Studies examining a combined population such as patients with either dyslipidemia or hypertension were not included unless the groups were analyzed separately. Where telephone calls were the primary intervention, the study was excluded as such an intervention could occur using landline telephones. Similarly, web-based interventions were not included as they could occur using a computer or tablet. Studies that recruited less than 10 subjects in each arm were excluded. Qualitative studies or those with no clinical endpoints were not included. Non-English language results were not included. Abstracts, case reports, editorials, and conference presentations were excluded.

### Data Extraction and Critical Appraisal

Article screening was performed by reviewing abstracts (by PI). Clinical outcome data were extracted from article text, tables, and figures independently by 2 researchers (PI and DT) from articles where it was available in the text, tables, figures, or supplementary material. Any discrepancies were resolved after the collaborative review. The final results were reviewed by all authors.

### Meta-Analysis

Meta-analysis was performed by combining event rates of dichotomous variables and using the supplied means and standard deviations for continuous variables. The odds ratio (OR) was used as a summary statistic for dichotomous variables. A random effect model was used. Chi-square tests were used to study heterogeneity between trials. The I^2^ statistic was used to estimate the percentage of total variation across studies due to heterogeneity rather than chance. An I^2^ value of greater than 50% was considered to represent substantial heterogeneity. Subgroup analyses were not possible due to the lack of patient-level data. All *P* values were two-sided. All statistical analyses were conducted with Review Manager Version 5.3 (Cochrane Collaboration, Software Update).

## Results

### Quantity and Quality of Studies

**Figure 1:**
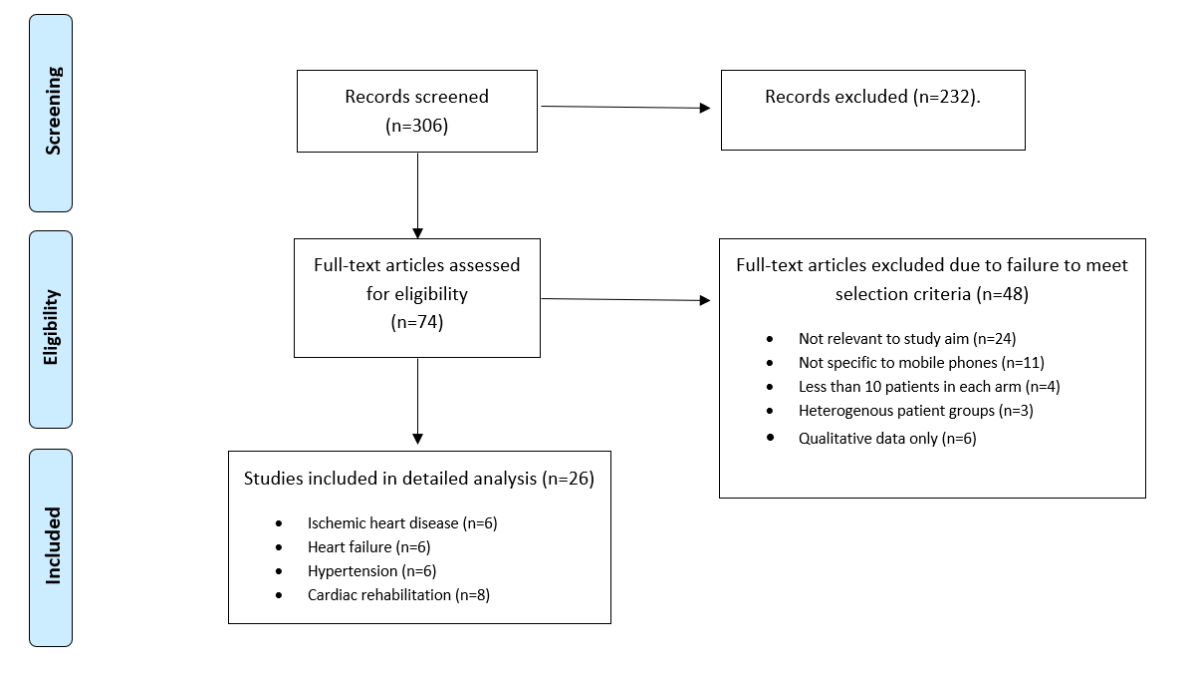
Search strategy and results for the Preferred Reporting Items for Systematic Reviews and Meta-Analyses (PRISMA) method.

Using the search strategy described earlier, 306 unique references were retrieved (465 before deduplication). The screening process is summarized in the PRISMA chart in Figure 1. A total of 26 references were included in the final systematic review, comprising a total of 6713 patients. These are summarized in Tables 1 and 2 and in Multimedia Appendices 1 and 2.

Of the 26 RCTs, the target population was ischemic heart disease in 6 studies [14-19], heart failure in 6 [20-25], hypertension in 6 [26-31], and CR in 8 [32-38]. Of these 8, 1 paper included 2 separate trials [35].

The studies were performed in 17 different countries, including 9 studies from Europe, 6 from North America, 5 from Australia/New Zealand, 4 from Asia, and 1 each from Africa and South America. In total, 10 studies examined a 1-way SMS intervention, 3 examined an interactive SMS intervention—where participants could reply, 4 examined an automatic telemonitoring system in which metrics were transmitted to the research team without the need for manual entry, 6 examined a manual telemonitoring system, and 3 studies examined a smartphone app that did not fit the previous criteria. Moreover, 10 studies included a *back-end*, whereby participants were able to transmit data that were viewable by the researchers or clinicians.

Blinding of the participants was not possible in any of the 26 identified studies. This was expected given the nature of the interventions. Only 8 studies featured blinding of the researchers or outcome assessors, and only 7 studies were adjudicated as having a low risk of bias (Multimedia Appendix 3).

**Table 1:** Characteristics of the studies included in the systematic review.

Reference	Country	Multicenter	Population	Number of patients (N)	Follow-up (weeks)	Intervention	Back-end
Blasco 2012 [14]	Spain	No	IHD^a^-ACS^b^	203	52	Manual telemonitoring	Yes
Chow 2015 [15]	Australia	No	IHD-ACS and stable CAD^c^	710	26	One-way SMS	No
Fang 2016 [16]	China	No	IHD-stable CAD	271	26	One-way SMS	No
Khonsari 2015 [17]	Malaysia	No	IHD-ACS	62	8	One-way SMS	No
Park 2015 [18]	United States	No	IHD-ACS, stable	90	4.5	Interactive SMS	No
Quilici 2013 [19]	France	No	IHD-ACS	499	4.5	One-way SMS	No
Chen 2019 [20]	China	No	HF^d^	512	26	One-way SMS	No
Dendale 2012 [21]	Belgium	Yes	HF	160	26	Auto telemonitoring	Yes
Koehler 2011 [22]	Germany	Yes	HF	710	52	Auto telemonitoring	Yes
Scherr 2009 [23]	Austria	No	HF	120	26	Manual telemonitoring	Yes
Seto 2012 [24]	Canada	No	HF	100	26	Auto telemonitoring	Yes
Vuorinen 2014 [25]	Finland	No	HF	94	26	Manual telemonitoring	Yes
Bobrow 2016 [26]	South Africa	No	HTN^e^	1372	52	Interactive SMS	No
Kiselev 2012 [27]	Russia	No	HTN	199	52	One-way SMS	No
Logan 2012 [28]	Canada	Yes	HTN	110	52	Auto telemonitoring	Yes
Morawski 2018 [29]	United States	Yes	HTN	411	12	Manual telemonitoring	No
Morikawa 2011 [30]	Japan	No	HTN	41	4	One-way SMS	No
Varleta 2017 [31]	Chile	Yes	HTN	314	26	One-way SMS	No
Bravo-Escobar 2017 [32]	Spain	No	CR^f^-ischemic CM^g^	28	8	App (other)	Yes
Del Rosario 2018 [33]	Australia	No	CR (mixed)	66	12	Manual telemonitoring	No
Maddison 2019 [34]	New Zealand	Yes	IHD-ACS	162	24	App (other)	Yes
Pandey 2017 [35]	Canada	No	CR-ACS	34	52	One-way SMS	No
Pandey 2017 [35]	Canada	No	CR-ACS	50	52	One-way SMS	No
Pfaeffli Dale 2015 [36]	New Zealand	Yes	CR-ACS and stable CAD	123	26	Interactive SMS	No
Piotrowicz 2010 [37]	Poland	No	CR (HF)	152	8	Manual telemonitoring	No
Varnfield 2014 [38]	Australia	No	CR-ACS	120	6	App (other)	Yes

^a^IHD: ischemic heart disease.

^b^ACS: acute coronary syndrome.

^c^CAD: coronary artery disease.

^d^HF: heart failure.

^e^HTN: hypertension.

^f^CR: cardiac rehabilitation.

^g^CM: cardiomyopathy.

**Table 2:** Endpoints examined in randomized trials of mobile phone technology in cardiovascular disease.

Author	Population	Primary endpoint	Primary result	Major secondary endpoints				
				Mortality	Hospitalization	Blood pressure	BMI	Medication adherence
Blasco 2012 [14]	IHD^a^	Cardiovascular risk	NS^b^	NR^c^	NR	NS	NS	NR
Chow 2015 [15]	IHD	Lipid profile	P^d^	NR	NR	P	P	NR
Fang 2016 [16]	IHD	Medication adherence	P	NR	NR	NR	NR	P1^e^
Khonsari 2015 [17]	IHD	Medication adherence	P	NS	NS	NR	NR	P1
Park 2015 [18]	IHD	Medication adherence	P	NR	NR	NR	NR	P1
Quilici 2013 [19]	IHD	Medication adherence	P	NR	NR	NR	NR	P1
Chen 2019 [20]	HF^f^	Mortality	NS	NS1^g^	P	NR	NR	P
Dendale 2012 [21]	HF	Mortality	P	P1	NS	NR	NR	NR
Koehler 2011 [22]	HF	Mortality	NS	NS1	NS	NR	NR	NR
Scherr 2009 [23]	HF	Mortality	NS	NS1	NS	NR	NR	NR
Seto 2012 [24]	HF	BNP^h^	NS	NR	NR	NR	NR	NR
Vuorinen 2014 [25]	HF	Readmissions	NS	NS	NS1	NR	NR	NR
Bobrow 2016 [26]	HTN^i^	Blood pressure	P	NR	NS	P1	NR	P
Kiselev 2012 [27]	HTN	Blood pressure	P	NR	NR	P1	NS	NR
Logan 2012 [28]	HTN	Blood pressure	P	NR	NR	P1	NR	NR
Morawski 2018 [29]	HTN	Blood pressure	NS	NR	NR	NS1	NR	P
Morikawa 2011 [30]	HTN	Blood pressure	P	NR	NR	P1	NS	NR
Varleta 2017 [31]	HTN	Medication adherence	NS	NR	NR	NS	NR	NS1
Bravo-Escobar 2017 [32]	CR^j^	Physical fitness	NS	NR	NR	NS	NS	NR
Del Rosario 2018 [33]	CR	CR completion rate	P	NR	NR	NS	NS	NR
Maddison 2019 [34]	CR	Physical fitness	NS	NR	NR	NR	NR	NR
Pandey 2017 [35]	CR	Medication adherence	NS	NR	NR	NR	NR	NS1
Pandey 2017 [35]	CR	Lifestyle adherence	NS	NR	NR	NR	NR	NR
Pfaeffli Dale 2015 [36]	CR	Lifestyle adherence	NS	NR	NR	NS	NS	P
Piotrowicz 2010 [37]	CR	Functional status	P	NR	NR	NR	NR	NR
Varnfield 2014 [38]	CR	CR completion rate	NS	NR	NR	NS	P	NR

^a^IHD: ischemic heart disease.

^b^NS: not significant.

^c^NR: not reported.

^d^P: positive.

^e^P1: positive primary endpoint.

^f^HF: heart failure.

^g^NS1: not significant primary endpoint.

^h^BNP: brain natriuretic peptide.

^i^HTN: hypertension.

^j^CR: cardiac rehabilitation.

### Description of Studies

#### Ischemic Heart Disease

A total of 6 interventions were identified that were targeted at patients with ischemic heart disease, excluding patients who were exclusively recruited from CR. The most commonly assessed primary endpoint was medication adherence, and the results are tabulated in Multimedia Appendix 4.

The largest and most comprehensive study was the pivotal Tobacco, Exercise and Diet Messages (TEXT ME) trial [15]. A total of 352 patients received 4 motivational text messages a week, randomly during daylight hours over a 6-month period. The messages focused on secondary prevention strategies. The primary endpoint was low-density lipoprotein cholesterol levels, which were lower in the intervention group (79 mg/dL [2.04 mmol/L] vs 84 mg/dL [2.17 mmol/L]; *P*=.04). Several other endpoints were examined, and significant improvements in systolic blood pressure (BP; −7.6 mm Hg; *P*<.001), physical activity, and smoking rates (88/339, 25.9% vs 152/354, 42.9%; *P*<.001) were noted. The majority of patients found the intervention motivational, educational, and useful. Medication adherence was not assessed.

A Spanish RCT investigated a 2-way messaging service in patients who had experienced an acute coronary syndrome [14]. Participants were provided with a mobile phone and a sphygmomanometer, a glucose meter, and a lipid meter. After measuring their results, they were entered into an app that transmitted the data to a cardiologist, who remotely monitored the data and responded with recommendations to the participant via text message. The content and nature of these messages were not described. Mean BMI improved in the intervention arm but did not change in the control arm (−0.37 [95% CI –0.08 to 0.04] vs 0.38 kg/m^2^ [95% CI –0.11 to 0.85]; *P*=.02). The proportion of patients meeting the BP target was higher in the intervention arm (63/101, 62.3% vs 44/102, 43.1%; *P*=.01), although there was no significant difference in BP between the 2 groups. Medication adherence was not assessed.

Fang et al [16] examined the use of SMS via both standard delivery and in combination with a messaging app called *Micro Letter,* and compared these results with standard care, in a 3-arm RCT [16]. Compliance was assessed using the Morisky Medication Adherence Scale (MMAS-4), and results were presented in a logistic regression analysis. Patients receiving messages via 2 platforms were more compliant than those just receiving standard SMS. Both intervention groups demonstrated superior compliance with the control group.

A small study performed in Malaysia randomized 62 patients with a recent acute coronary syndrome to receive an SMS reminder before scheduled medication times or usual care only, for an 8-week period post discharge [17]. Patients were more likely to have *high compliance*, as measured by a score of 8 on the MMAS-8 scoring scale (20/31, 65% vs 4/31, 13%; *P*<.001).

Park et al [18] randomized patients to receive SMS reminders and education, SMS education only, and usual care. Electronic pill bottles, along with the MMAS score, were used to evaluate compliance. The percentage of correct doses taken for antiplatelets over 30 days was higher in the intervention groups than in the control groups (88% and 87% vs 72%; *P*=.04). However, no significant difference was observed for compliance with statin therapy.

Quilici et al [19] performed a randomized trial of 499 patients to receive daily text messages or standard care and assessed aspirin adherence based on patient interview data and also platelet function. The self-reported rates of nonadherence were significantly different between the intervention group and the control group in both the self-reported (7/250, 2.8% vs 18/249, 7.2%; *P*=.02) and the platelet function testing endpoints (13/250, 5.2% vs 28/249, 11.2%; *P*=.01).

#### Cardiac Failure

A total of 6 RCTs assessed the efficacy of telemedicine-based interventions in the management of heart failure; 5 out of the 6 studies demonstrated at least one clinical benefit for the intervention, although there was no endpoint that was shown to be consistently improved across all studies. One study demonstrated an improvement in mortality [21]. The results are summarized in Multimedia Appendix 5.

A recent large RCT from China randomized patients with chronic heart failure into 3 arms: structured telephone support (a single phone call within 30 days of discharge with the opportunity to speak to a nurse during work hours), an SMS-based support system, or a control group [20]. The SMS system consisted of daily educational messages for 10 days and weekly reminder messages thereafter. These messages were automated, not personalized, and could not be replied to. A comparison of the SMS group with the control group demonstrated significantly lower readmission rates as well as higher rates of medication compliance. There was no significant difference in mortality or quality of life. It was not specified how rates of medication noncompliance were measured.

Dendale et al [21] conducted a multicenter study in Belgium that compared patients who were established on an automated monitoring platform including BP, heart rate, and weight with those receiving standard care [21]. Alerts were received by the patient’s general practitioner when abnormal parameters were encountered on 2 consecutive days. Despite its small sample size, a statistically significant difference in all-cause mortality was observed (4/80, 5% vs 14/80, 17%; *P*=.01).

The study by Koehler et al [22] comprised 710 patients with severely reduced left ventricular ejection fraction (LVEF) who were randomized 1:1 to receive 24-hour home telemonitoring or usual care. Devices for electrocardiogram (ECG), BP, and body weight were paired via Bluetooth to a personal digital assistant, which transmitted the data via a mobile phone service to a central data monitoring unit. Patients were followed up for a minimum of 12 months (mean 26 months). There was no significant difference in mortality (15% in both groups, 54/354 intervention vs 55/356 in the control group) or hospitalizations, although patients in the intervention arm may have had better quality of life as evidenced by higher SF-36 scores.

The MOBIle TELemonitoring in Heart Failure Patients study compared the use of a manual monitoring system with standard care [23]. Patients were given a mobile phone and asked to measure their weight and BP daily. The results were manually entered into the mobile phone app. The physicians received alerts for measurements that were abnormal. Patients in the intervention group had lower rates of hospitalization, and functional status improved by one New York Heart Association (NYHA) score. When patients were hospitalized, the median length of stay in the intervention group was also shorter than that of the control group (6.5 vs 10 days; *P*=.04). Of note, this trial was prematurely terminated due to a high rate of technical difficulties; 12 out of 54 patients (22%) in the intervention arm were unable to operate the app.

Seto et al [24] recruited 100 patients who were randomized into telemonitoring and control groups. The patients in the telemonitoring group performed BP and weight measurements, which were transmitted via Bluetooth and a smartphone to a data monitoring unit. Patients were required to answer a daily symptom-based questionnaire. Alerts of varying priorities were sent to the treating clinician based on the results. Quality of life scores were improved in the telemonitoring group (Minnesota Living With Heart Failure Questionnaire [MLHFQ]), but there was no difference in overall mortality, hospitalization, or ejection fraction.

Vuorinen et al [25] studied a Finnish cohort of patients with severe heart failure with reduced ejection fraction (<35%), who were symptomatic with a NYHA score of 2 or more, and known to the established outpatient heart failure service. Patients were provided with a mobile phone as well as a sphygmomanometer and a weight scale, and they entered these results via an app, which also contained a symptomatology questionnaire. Results were entered once a week. The authors noted no difference in days spent in hospital for admission and no difference in N-terminal prohormone of brain natriuretic peptide concentrations or LVEF. There was a significantly higher use of resources for patients in the intervention arm, specifically with regard to the number of visits to the clinic.

#### Hypertension

In total, 6 RCTs examined smartphone apps in the management of patients previously diagnosed with hypertension; 4 of these demonstrated a statistically significant reduction in systolic BP. The results are summarized in Multimedia Appendix 6.

The SMS Text Adherence Support trial randomized 1372 patients to receive SMS information, interactive SMS, or usual care [26]. The follow-up period was 12 months. Patients receiving the information only experienced a mean systolic BP reduction of 2.2 mm Hg (95% CI −4.4 to −0.04, *P*=.04). There was no significant difference in the group that received interactive SMS. Patients in the SMS group were also more likely to be adherent to their medications, with adherence defined as medications correctly taken on over 80% of days (62.1% vs 49%; *P*<.001 for both groups).

Kiselev et al [27] performed a randomized study of 199 patients. The intervention group received SMS reminders to promote medication compliance as well as healthy behaviors, and the control group received standard care. BP fell significantly in both groups; however, the difference was more marked in the intervention group (23.7 mm Hg vs 6.9 mm Hg; *P*=.04). Of note, the baseline BP was significantly higher in the control group, suggesting problems with randomization. Nearly 77% (47/62) of the patients in the intervention group achieved their goal BP. There was a significant dropout rate in the intervention arm, with only 62 of 97 (63.9%) participants attending the 12-month follow-up.

A multicenter Canadian study of 110 diabetic patients examined the impact of an automatic telemonitoring system using a smartphone app and Bluetooth-enabled BP machine [28]. Abnormal readings were responded to by an automated system, which transmitted self-care messages to the patient. Clinicians were alerted only to highly abnormal values. The control group received the same BP machine, but without any intervention from the automated system. The mean daytime ambulatory systolic BP decreased significantly in the intervention group by 9.1 (SD 5.6) mm Hg (*P*<.001) and did not significantly change in the control group.

The Medication Adherence Improvement Support App For Engagement—Blood Pressure app used a web-based recruitment platform to randomize 411 patients with essential hypertension to receive either an automatic, Bluetooth-enabled sphygmomanometer or the companion Medisafe smartphone app [29]. The app incorporated medication reminders after either the user had input their medication list or it was auto-populated via an electronic medical record. Participants were followed up for 12 weeks. Medication adherence, as determined by the MMAS-8 questionnaire score, increased by 0.4 points in the intervention group compared with the control group. This result was statistically significant, but not thought to be clinically significant, as the authors targeted an MMAS-8 score improvement of 2.0 to be a meaningful improvement. There was no significant difference in BP.

Morikawa et al [30] studied the effect of minimizing salt intake on BP in a group of hypertensive railroad employees. A total of 41 patients were allocated, via a quasi-randomized process, to an intervention or control group. Those in the intervention group were asked to measure urine sodium concentration via a salt sensor and container provided by the investigators. The results were then used to trigger a personalized email, delivered via mobile phone, to the participants. BP and salt intake were assessed over 4 weeks. The intervention group had a larger mean reduction in systolic BP (5.4 mm Hg vs 2.2 mm Hg), although this did not reach statistical significance. Diastolic BP, however, fell significantly (6.2 mm Hg vs 1.6 mm Hg; *P*=.01). The intervention was shown to increase the number of patients modifying their dietary salt intake.

A multicenter study from Chile examined the effect of 1 SMS every 2 weeks on BP and medication adherence [31]. There was no significant difference for either of these endpoints, although there was a very high dropout rate, meaning over 43.3% (136/314) of participants did not have their BP measured at the completion of the study.

#### Cardiac Rehabilitation

Eight randomized controlled trials studied the addition of mobile phone interventions (MPIs) to standard CR. All trials demonstrated at least one benefit in the intervention group, although specific positive results varied significantly between trials.

Del Rosario et al [33] randomized patients undergoing home-based or hospital-based CR for the first time to receive a sphygmomanometer and weighing scale that could transmit data via a mobile phone using near field communication technology as an adjunct to standard CR or standard CR alone. The authors noted an improvement in CR completion rates within the intervention arm (27/33, 88% vs 20/33, 67%; *P*=.04).

The Text4Heart trial, undertaken in New Zealand, examined the addition of a personalized 24-week program of educational and motivational text messages, delivered daily, to standard CR for patients with postmyocardial infarction [36]. Apart from self-reported medication compliance at 6 months, there was no significant difference between other self-reported endpoints at the completion of 6-month follow-up.

Piotrowicz et al [37] examined the utility of a mobile phone paired with a 3-lead ECG monitor in patients with cardiac failure. Patients were randomized to receive home telemonitoring for CR or standard CR and were followed up over an 8-week period. Patients in the home telemonitoring group answered questions regarding symptomatic status before a session of CR and transmitted their ECG to a remote center. Once reviewed, the patients were given approval to commence the session. ECGs were automatically transmitted during the session and at the conclusion of the session, with the patient able to transmit an ECG if they experienced chest pain or any other concerning symptoms. Although clinical endpoints were similar, there was a 20% dropout rate in the standard CR group, predominantly driven by patients being unable to afford the costs of attending CR and due to difficulties attending CR due to time constraints.

Varnfield et al [38] compared a home-based CR service that was delivered by a smartphone with standard CR in patients following an acute coronary syndrome. The components of the program included a step-counter, a* wellness diary*, weekly teleconferences with a mentor, motivational text messages, and videos. Uptake rates in the intervention group were significantly better (48/60, 80% vs 37/60, 62%; *P*=.04); however, there were no significant differences in clinical outcomes over 6 weeks.

Pandey et al [35] published an RCT of 50 postmyocardial infarction patients who received 4 SMS messages daily reminding them to exercise. The patients kept a logbook of the days they exercised and the duration. According to this self-reported endpoint, patients in the intervention arm exercised more frequently (17 days per 12.5 hours per month vs 13 days per 8.5 hours per month). There was, however, no difference in cardiorespiratory fitness as measured by metabolic equivalents during a Bruce protocol exercise stress test. In the same paper was a separate RCT of 34 patients with recent myocardial infarction attending CR receiving daily SMS medication reminders [35]. Only patients taking once-daily medications were included in the trial, which introduces selection bias, as patients taking once-daily medications may have less difficulty adhering to their prescribed regimen compared with those requiring multiple daily dosing. The messages were generic, such as *please remember to take your morning medications now* and did not specify any information about the patient or the medications themselves. The patient was not able to reply to the message. Medication compliance was assessed using logbooks kept by the patient. The compliance rate, as measured by days where medications were all taken, was significantly higher in the intervention group (94% vs 80%; *P*<.001).

Two studies focused on the use of a wearable ECG monitoring system using smartphone technology. An RCT from New Zealand compared home-based CR using a smartphone-based platform including an ECG monitoring vest and web-based education with center-based CR. This noninferiority trial demonstrated comparable average physical fitness (as measured by maximal oxygen consumption: VO_2_ max) between the 2 groups [34]. Preliminary results from a single center in a Spanish study revealed no significant differences in exercise-related outcomes between a group of patients with ischemic cardiomyopathy who utilized a wearable ECG vest with a smartphone connection for home-based CR and those undergoing a traditional hospital-based program [32].

### Meta-Analysis

**Figure 2:**
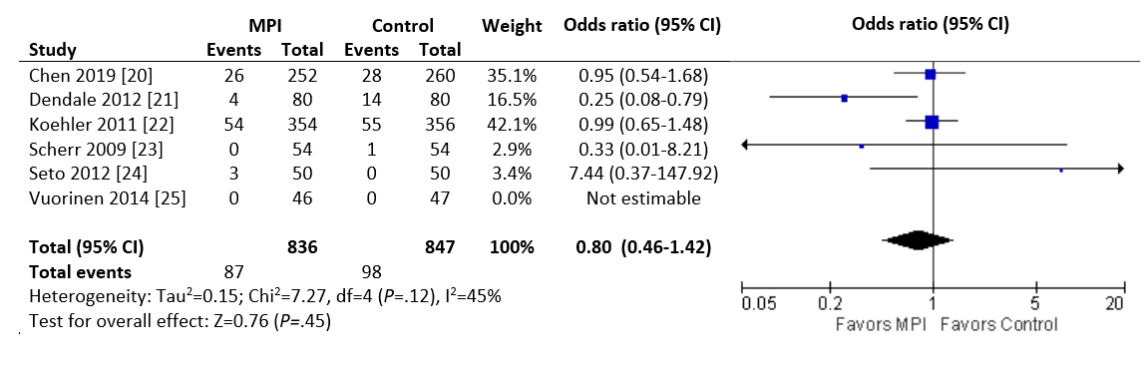
Forest plot of the odds ratio (OR) of mortality in patients with heart failure who were involved in randomized controlled trials comparing a mobile phone intervention versus control. The estimate of the OR of each trial corresponds to the middle of the squares, and the horizontal line shows the 95% CI. The summary OR is represented by the middle of the solid diamond. A test of heterogeneity is given below the summary statistics. MPI: mobile phone intervention, df: degrees of freedom.

For the heart failure cohort, all 6 studies were included in the meta-analysis. There was no significant difference in mortality (measured at 6 months in all studies, with the exception of Koehler et al [22], who reported mortality at 12 months). The mortality rate in the intervention group was 10.4% compared with 11.6% in the control group (87/836 vs 98/847; *P*=.45; I^2^=45%; Figure 2).

**Figure 3:**
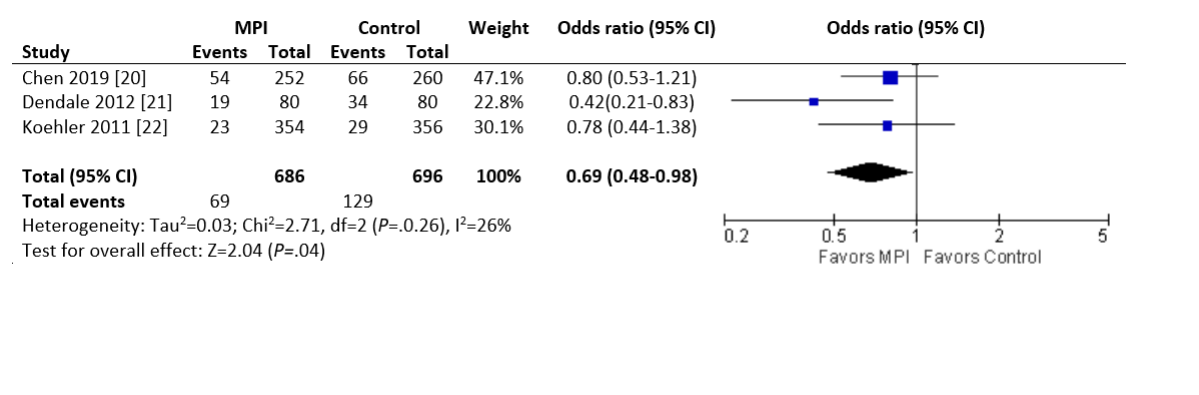
Forest plot of the odds ratio (OR) of heart failure readmissions in patients with heart failure who were involved in randomized controlled trials comparing a mobile phone intervention versus control. The estimate of the OR of each trial corresponds to the middle of the squares, and the horizontal line shows the 95% CI. The summary OR is represented by the middle of the solid diamond. A test of heterogeneity is given below the summary statistics. MPI: mobile phone intervention, df: degrees of freedom.

Readmissions due to heart failure over 6 months were less common in the intervention group than in the control group for the 3 studies that reported this endpoint (96/686, 14.0% vs 129/696, 18.5%; OR 0.69, 95% CI 0.48 to 0.98; *P*=.04; I^2^=26%; Figure 3).

**Figure 4:**
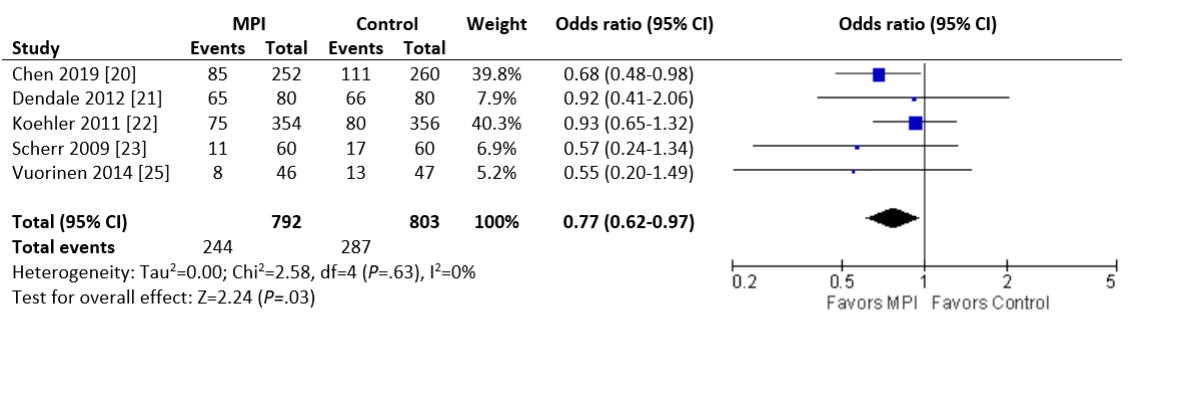
Forest plot of the odds ratio (OR) of all-cause readmissions in patients with heart failure who were involved in randomized controlled trials comparing a mobile phone intervention versus control. The estimate of the OR of each trial corresponds to the middle of the squares, and the horizontal line shows the 95% CI. The summary OR is represented by the middle of the solid diamond. A test of heterogeneity is given below the summary statistics. MPI: mobile phone intervention, df: degrees of freedom.

The rate of hospitalization for any reason over 6 months was significantly lower in the intervention group (244/792, 30.8% vs 287/803, 35.7%; OR 0.77, 95% CI 0.62 to 0.97; *P*=.03; I^2^=0%; Figure 4).

**Figure 5:**
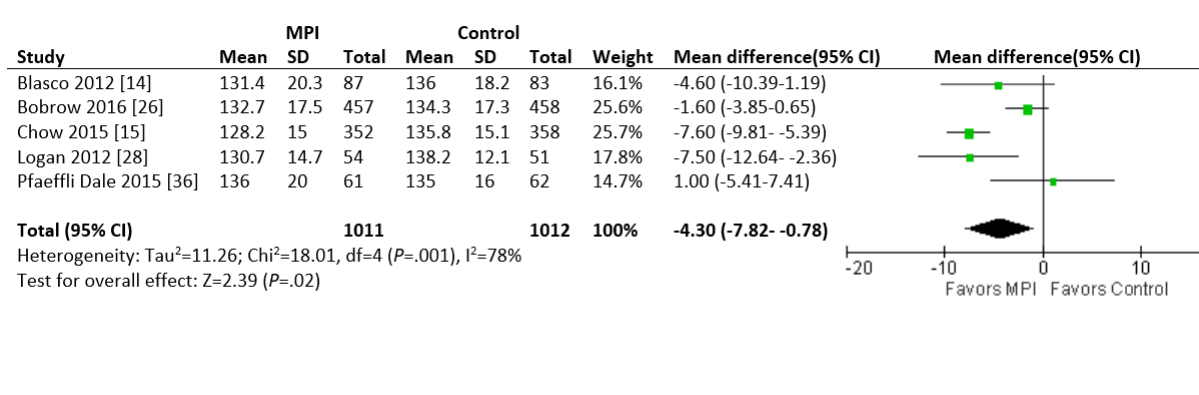
Forest plot of the mean difference in blood pressure in patients with hypertension who were involved in randomized controlled trials comparing a mobile phone intervention versus control. The mean difference of each trial corresponds to the middle of the squares, and the horizontal line shows the 95% CI. The summary mean difference is represented by the middle of the solid diamond. A test of heterogeneity is given below the summary statistics. MPI: mobile phone intervention, df: degrees of freedom.

The difference in systolic BP was analyzed from 5 studies that reported the endpoint at 6 or 12 months. The mean systolic BP was 4.3 mm Hg less in the intervention group than in the control group (95% CI −7.8 to −0.78 mm Hg; *P*=.02; Figure 5). Substantial heterogeneity was identified (I^2^=78%). Two studies that reported the endpoint were excluded from analysis, one due to a significantly different BP at baseline between the 2 groups [27] and one due to a very high withdrawal rate of over 40% [31].

**Figure 6:**
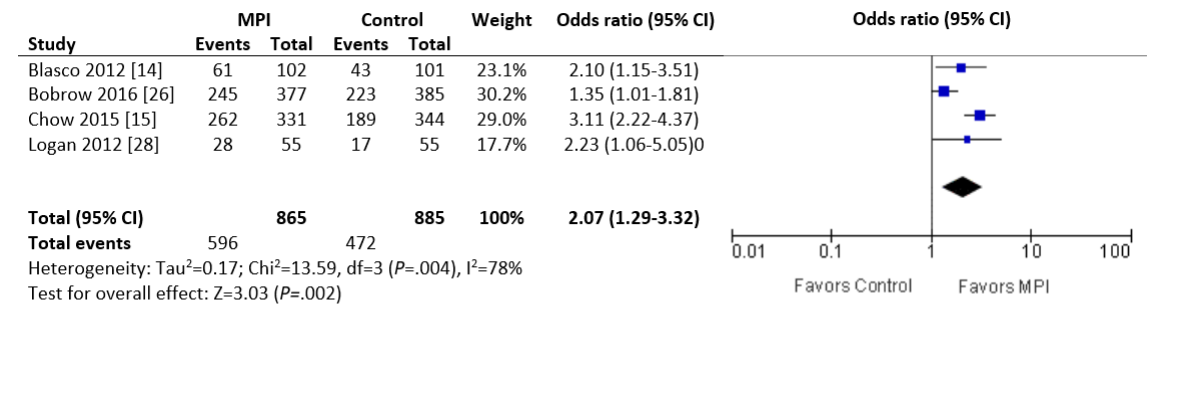
Forest plot of the odds ratio (OR) in patients with hypertension who achieved the prespecified target blood pressure and who were involved in randomized controlled trials comparing a mobile phone intervention versus control. The estimate of the OR of each trial corresponds to the middle of the squares, and the horizontal line shows the 95% CI. The summary OR is represented by the middle of the solid diamond. A test of heterogeneity is given below the summary statistics. MPI: mobile phone intervention, df: degrees of freedom.

Four studies reported the percentage of patients who reached the target BP, defined as 140/90 mm Hg in 3 studies [14,25,26] and 130/80 mm Hg in the other [28]. In the meta-analysis, patients in the intervention arm were more likely to achieve their target BP than those in the control group (596/865, 68.9% vs 472/885, 53.3%; OR 2.07, 95% CI 1.29 to 3.32; *P*=.002; I^2^=78%; Figure 6).

There was no significant difference in the change in BMI between the 4 studies that reported the endpoint after 6 or more months (mean difference −0.46; 95% CI −1.44 to 0.52; *P*=.36; I^2^=82%; Multimedia Appendix 7).

A meta-analysis of medication adherence could not be performed, as there was no uniform measurement for assessing the outcome.

## Discussion

### Principal Findings

This meta-analysis demonstrated that in patients with heart failure, the use of MPIs reduced the rate of hospital admission, both in relation to total admissions and heart failure admissions. There was no significant difference in mortality rates between the groups. In patients with hypertension, those who used MPIs had a significantly lower systolic BP and were more likely to reach the target BP. There was no significant difference in BMI.

Mobile phone and smartphone technology represent a significant opportunity for health care providers to improve outcomes for large populations of patients with CVD. Although no single holistic cardiac care app has been rigorously trialed, a multitude of small, targeted apps have been studied. In general, these heterogeneous and underpowered data do not allow for clear conclusions to be made on the overall benefit of MPIs; however, several important observations are made.

In patients with ischemic heart disease, MPIs universally improved medication compliance. It was not possible to perform meta-analysis of this endpoint due to the variation in reporting systems used between studies, but this is an important finding. Reasons for noncompliance are multiple; however, by providing physical reminders as well as motivational support, text messaging appears to be an effective method of reinforcing adherence, particularly in the context of asymptomatic disease. Given the relatively low cost and negligible risk of text messaging, it could be considered a mainstream management strategy for patients possessing a mobile phone. It remains to be seen, however, whether improved compliance leads to a clinically significant benefit, as cardiovascular event rates were generally not examined in these trials. The successful use of mobile phone technology to promote medication compliance has also been demonstrated in other fields of medicine [24], with one recent meta-analysis estimating an improvement in patient compliance from 50% to 67.8% with consistent use of text message reminders for patients with chronic disease [39,40].

In hypertensive patients, reductions in BP are also likely a reflection of improved compliance [26]. In addition, apps that emphasize positive lifestyle modifications such as dietary improvement and increased exercise regularity are likely to provide an impetus for nonpharmacological reduction in BP, as was seen in trials aimed at the general population [41,42]. Reduction of BP, and improvement in compliance, particularly in relation to antiplatelet agents following revascularization, is likely to reduce long-term recurrence rates of ischemic heart disease; however, none of the studies examined a follow-up period beyond 6 months. Furthermore, all studies examining SMS compared SMS with a control arm. Different types of SMS (personalized vs generic and interactive vs nonreply) were not compared with each other and neither were variations in SMS frequency (eg, daily vs multiple times daily). Although the body of evidence showing the benefits of SMS interventions for compliance against a control group is growing, the optimal nature and frequency of SMS has not been established and should be the focus of future studies. The majority of studies reviewed here relied on self-reported measures of compliance or compliance questionnaires, both of which are subjective. A reduction in systolic BP and an increased likelihood of reaching target BP was observed in the meta-analysis, although substantial heterogeneity contributed to this result, due to the variety of patient populations and interventions considered.

In the cardiac failure cohort, five studies used home monitoring of BP and weight. Although benefits were shown with regard to quality of life and functional status, only one study demonstrated a difference in mortality, and the results of the meta-analysis were negative. The negative result for mortality was driven largely by the study by Koehler et al [22], which used remote monitoring of ECG, weight, and BP in combination with a 24-hour physician monitoring service. The authors recruited a cohort of patients with severe cardiomyopathy and ejection fractions of <30% [22]. These patients had a poor prognosis, as evidenced by the 15% mortality rate at the end of the 26-month follow-up period. It is possible that the severity of cardiomyopathy in these patients meant that despite remote monitoring, readmissions and mortality were unavoidable in such a cohort. In addition, it is not clarified what the triggers and frequency for contacting patients were. Seto et al [24] utilized a similar model using a mobile phone and alerts to clinicians when the results were abnormal. This study of 100 patients was underpowered to detect any statistically significant reductions in mortality or hospitalization rates. Vuorinen et al [25] also studied a cohort of patients with severely impaired left ventricular function. The authors concluded that the intervention had a net negative benefit, as there were no improvements in clinical outcomes, and an increase in clinic visits for those patients. Patients in this study, however, only had measurements performed weekly. Heart failure with preserved ejection fraction was largely underrepresented in all these studies.

From these data, it is clear that not all interventions are equal. Dendale et al [21] demonstrated an intervention that improved both mortality and the rate of heart failure readmissions. This intervention used daily automatic data transmission, an alert-based system to *flag* patients with abnormal parameters, and involved the care of the patient’s general practitioner. All these components appear to be beneficial. Automatic data transmission, rather than manual data entry, eases the work burden on the patient and may improve compliance with the system. Although weekly data entry, as used in the study by Vuorinen et al [25], eases the compliance burden on the patient, it may not be sensitive enough to detect early decompensation. Using an alert-based system helps to identify patients who need closest monitoring and avoid data saturation of clinicians. Utilizing the expertise of a general practitioner who is familiar with the patient is also likely to be beneficial in optimizing therapy. Similarly, the study by Logan et al [28] in hypertensive patients used an automatic BP transmission process, which alerted the patient’s usual primary care clinician to abnormal values. This particular intervention proved 1 of 2 interventions that demonstrated a statistically significant BP reduction over at least 6 months. Therefore, it would appear that an optimal telemonitoring MPI for cardiovascular patients should include (1) collection of patient data using automatic methods rather than manual entry; (2) an automated back-end that will filter and identify abnormal data; (3) the input of the patient’s regular clinician; (4) an educational component, perhaps by text messaging; and (5) ease of use and limited technical issues.

The results for mobile phone technology as an adjunct to CR suggested potential improvements in medication adherence [36], participation rates [37-39], and quality of life scores [43]. Smartphone-based CR allows patients to participate from home, which is desirable, particularly for participants who may find it inconvenient or costly to attend hospital-based programs.

Other studies of telemonitoring in heart failure, using technologies other than mobile phones, have shown mixed results. Multiple meta-analyses have shown superior outcomes for telemonitoring in heart failure patients compared with standard care [44-46], although 2 large individual trials have been negative [47,48]. A recent meta-analysis included 37 RCTs with 9582 heart failure patients [46]. The authors identified a reduction in all-cause mortality for telemedical interventions compared with usual care (relative risk 0.81; 95% CI 0.70 to 0.94; I^2^=0.16). The methodologies and delivery of these interventions were highly variable, and the vast majority (32/37) did not use mobile phone–based systems. Examples of delivery methods include videoconference, telephone calls, websites, and purpose-built telemedical units capable of transmitting data. A head-to-head comparison of telemedical delivery methods has not been performed.

The cost-effectiveness of mobile phone technology for any of the aforementioned indications in CVD has not been conclusively studied. It is believed that a reduction of adverse clinical outcomes and an associated reduction in costs of hospitalization would likely offset costs of implementing the software and monitoring data, although there was a notable paucity of cost-effectiveness data. There remains only a single published cost-effectiveness analysis of an MPI based on randomized trial data [49]. The authors extrapolated the results of the clinical endpoints in the TEXT ME trial to a hypothetical cohort of 50,000 patients in a Markov model. They estimated cost savings of over Aus $10 million (US $6.53 million) in such a model.

Assuming that the mobile phone is not operated during driving or other dangerous tasks, there are no significant risks to the patient in any of the described interventions. There are several limitations of the available data contained within this systematic review and meta-analysis, and the results should be interpreted with caution. Although all studies were RCTs, they were generally small, with varying methodologies, and prone to bias. No follow-up period was longer than 26 months; thus, data on recurrent clinical events in the medium to long term were lacking. In addition, patient compliance with the mobile phone technology itself over longer periods is unknown. The utility of such interventions in the older population is uncertain, given that advanced age is one of the most significant risk factors for all forms of CVD. One study demonstrated a high rate of dropout due to inability of patients to operate the app (22%) [23], whereas another study reported a lower rate of unsuccessful app use (10%) recruited patients from online communities [29], which introduces a degree of selection bias as patients accessing these communities are likely to be more familiar with mobile phone technologies. Compliance data are typically measured using questionnaires and are thus prone to recall bias.

Several gaps remain in this literature. There is significant potential of this technology to gather data that can be reviewed in real-time and subsequently allow for rapid modifications in patient therapy in response, although this was only examined in a small number of trials. When patients are reviewed routinely by a clinician in the community, the clinician only sees a *snapshot* of the patient’s current health status. Trends in data such as weight and BP can only be compared between visits, rather than on a daily or regular basis. The connectivity of smartphone devices allows for data to be transmitted to a clinician who may be able to interpret them and provide management advice even from remote locations, thus allowing appropriate disease management to be instigated before the patient’s presentation with an acute medical event. It would appear that there is a large scope for mobile phone technology in this setting, and further study is needed to identify the optimal characteristics of such an app or program. Second, the impact of usability on the success of the MPIs was not adequately addressed in trials, with minimal reporting of this endpoint. We would suggest that any publication of an RCT describing an MPI have some evaluation of usability, as this will almost certainly influence compliance and hence the efficacy of the intervention, perhaps even more so than the capabilities of the MPI itself. Finally, the longest minimum follow-up period for any heart failure study was 12 months. A longer duration of monitoring in the studies may have allowed for a difference in readmission rates and, in particular, mortality to have been identified. Given that heart failure is a chronic condition, the benefits of an MPI may not be observed until several years of follow-up have occurred. As telehealth is a rapidly evolving field, it is surmised that studies are published with a short follow-up period to avoid publication at a time where the intervention is outdated or obsolete.

The majority of smartphone apps examined here had a single aim or function, and the management of CVD entails the optimization of multiple factors. Therefore, there remains a need for an adequately powered RCT examining the effect of a holistic smartphone intervention with multiple features that possess the ability to react to collected data and improve therapy and therefore clinical endpoints, and there is a need to identify which patients would benefit the most.

### Conclusions

MPIs have been applied to a variety of target groups in CVD. These fall into several categories including SMS apps, automatic and manual monitoring, and purpose-built apps. A number of RCTs have been published. The results suggest that mobile phone technology may improve medication adherence in patients with ischemic heart disease, BP in individuals with hypertension, and hospitalization rates in patients with heart failure. Further large RCTs with longer follow-up periods and a greater focus on clinical endpoints are required. However, given the relatively low risk and cost of such interventions, they should be considered as an adjunctive therapy in the management of patients with CVD or at risk of CVD.

## Appendix

Multimedia Appendix 1 Study interventions and target populations.

Multimedia Appendix 2 Other secondary endpoints of included studies.

Multimedia Appendix 3 Bias assessment according to the Cochrane Risk of Bias Tool.

Multimedia Appendix 4 Summary of mobile phone interventions for medication adherence in patients with ischaemic heart disease.

Multimedia Appendix 5 Mobile phone interventions for treatment of heart failure.

Multimedia Appendix 6 Mobile phone interventions for treatment of hypertension.

Multimedia Appendix 7 Forest plot of the mean difference in body mass index (BMI) in patients who were involved in randomised controlled trials comparing a mobile phone intervention (MPI) versus control. The mean difference of each trial corresponds to the middle of the squares, and the horizontal line shows the 95% confidence interval (CI). The summary mean difference, is represented by the middle of the solid diamond. A test of heterogeneity is given below the summary statistics where df refers to degrees of freedom.

## Abbreviations

BP: blood pressure
CR: cardiac rehabilitation
CVD: cardiovascular disease
ECG: electrocardiogram
LVEF: left ventricular ejection fraction
MMAS: Morisky Medication Adherence Scale
MPI: mobile phone intervention
NYHA: New York Heart Association
OR: odds ratio
PRISMA: Preferred Reporting Items for Systematic Reviews and Meta-Analyses
RCTs: randomized control trials
