# Supplementary material for: Mobile Phone Technologies in the Management of Ischemic Heart Disease, Heart Failure, and Hypertension: Systematic Review and Meta-Analysis
Source: JMIR Mhealth Uhealth. 2020 Jul 6;8(7):e16695. doi: 10.2196/16695 (PMC7381017; doi:10.2196/16695)
Supplement: Multimedia Appendix 1 [file mhealth_v8i7e16695_app1.docx]

|  | **Ischaemic Heart Disease** | **Heart Failure** | **Hypertension** | **Cardiac Rehabilitation** |
| --- | --- | --- | --- | --- |
| **SMS** | \| Chow \| \| --- \| \| Fang  Khonsari Quilici \| \| Park \| | Chen | \| Bobrow \| \| --- \| \| Kiselev \| \| Morikawa \| \| Varleta \| | Pandey Pfaeffli Dale |
| **Telemonitoring** | Blasco* | \| Dendale* \| \| --- \| \| Koehler* \| \| Scherr* \| \| Seto* \| \| Vuorinen* \| | \| Logan* \| \| --- \| \| Morawski \| |  |
| **Other smartphone application** |  |  |  | Bravo-Escobar*  Varnfield*  Maddison* |

*Contains a back-end for the clinician to respond to; SMS – short message service
