# Supplementary material for: Mobile Phone Technologies in the Management of Ischemic Heart Disease, Heart Failure, and Hypertension: Systematic Review and Meta-Analysis
Source: JMIR Mhealth Uhealth. 2020 Jul 6;8(7):e16695. doi: 10.2196/16695 (PMC7381017; doi:10.2196/16695)
Supplement: Multimedia Appendix 2 [file mhealth_v8i7e16695_app2.docx]

| **Author** | **Year** | **Primary Endpoint** | **Positive Secondary Endpoints** | **Negative Secondary Endpoints** |
| --- | --- | --- | --- | --- |
| ***Ischemic Heart Disease*** | | | | |
| Blasco | 2012 | CV Risk | - | CS,GP,LP |
| Chow | 2015 | Lipid profile | CS,LA,WC | - |
| Fang | 2016 | Medication adherence | - | - |
| Khonsari | 2014 | Medication adherence | FS | - |
| Park | 2015 | Medication adherence | - | - |
| Quilici | 2013 | Medication adherence | - | - |
| ***Heart Failure*** | | | | |
| Chen | 2019 | Mortality | LA | - |
| Dendale | 2012 | Mortality | - | - |
| Koehler | 2011 | Mortality | - | LOS,QOL |
| Scherr | 2009 | Mortality | - | FS,LVFn |
| Seto | 2012 | BNP | QOL | - |
| Vuorinen | 2014 | Readmissions | - | BNP,LVFn,QOL |
| ***Hypertension*** | | | | |
| Bobrow | 2016 | Blood pressure | - | QOL |
| Kiselev | 2012 | Blood pressure | - | CS |
| Logan | 2012 | Blood pressure | - | - |
| Morawski | 2018 | Blood pressure | - | - |
| Morikawa | 2011 | Blood pressure | - | WC |
| Varleta | 2017 | Medication adherence | - | - |
| ***Cardiac Rehabilitation*** | | | | |
| Bravo-Escobar | 2019 | Physical Fitness | - | LP,GP |
| Del Rosario | 2018 | CR Completion Rate | - | - |
| Maddison | 2018 | Physical Fitness | - | WC |
| Pandey | 2017 | Medication adherence | LA | LA |
| Pandey | 2014 | Lifestyle adherence | - | - |
| Pfaeffli Dale | 2015 | Lifestyle adherence | - | - |
| Piotrowicz | 2010 | Functional Status | PF | QOL |
| Varnfield | 2014 | CR Completion Rate | QOL | GP,LP,PF,WC |

BNP- Brain natriuretic peptide; CS – cigarette smoking; FS – Functional Status; GP – glycaemic profile, LA – lifestyle adherence, LVFn – left ventricular function, LP – lipid profile, PF – physical fitness, QOL – quality of life, WC – waist circumference
