# Supplementary material for: Mobile Phone Technologies in the Management of Ischemic Heart Disease, Heart Failure, and Hypertension: Systematic Review and Meta-Analysis
Source: JMIR Mhealth Uhealth. 2020 Jul 6;8(7):e16695. doi: 10.2196/16695 (PMC7381017; doi:10.2196/16695)
Supplement: Multimedia Appendix 3 [file mhealth_v8i7e16695_app3.docx]

**Table 3:**

| **Author** | **Year** | **Random-isation** | **Complete- ness of Data** | **Outcome Measurement** | **Reporting** | **Overall** | **Specific Limit-ations** |
| --- | --- | --- | --- | --- | --- | --- | --- |
| ***Ischaemic Heart Disease*** | | | | | | | |
| Blasco | 2015 | S | S | L | L | S | High drop out rate (16%) |
| Chow | 2015 | L | L | L | L | L |  |
| Fang | 2016 | L | L | L | L | L | SMS only but still required a smartphone |
| Khonsari | 2014 | S | L | L | L | S | Small sample size (n = 62) |
| Park | 2015 | L | L | L | L | L | Small sample size (n = 90) |
| Quilici | 2013 | S | L | L | S | S | Randomisation process not clearly defined |
| ***Heart Failure*** | | | | | | | |
| Chen | 2019 | L | L | S | L | S | Highly reliant on self-reported data |
| Dendale | 2012 | L | L | S | L | S |  |
| Koehler | 2011 | L | L | L | L | L |  |
| Scherr | 2009 | S | S | L | L | S | 22% were unable to operate the application |
| Seto | 2012 | L | S | S | L | S | Seasonal bias possible |
| Vuorinen | 2014 | L | L | L | S | L |  |
| ***Hypertension*** | | | | | | | |
| Bobrow | 2016 | L | S | L | L | L | 8.5% drop-out rate |
| Kiselev | 2012 | H | S | S | L | H | Blood pressure unequal between groups |
| Logan | 2012 | L | L | L | S | S | Control group compliance was unknown |
| Morawski | 2018 | L | L | L | L | S | Online recruitment platform – selection bias |
| Morikawa | 2011 | S | L | L | L | S | Limited to employees of a certain company |
| Varleta | 2017 | L | S | L | L | S | High drop out rate (44%) |
| ***Cardiac Rehabilitation*** | | | | | | | |
| Bravo-Escobar | 2019 | S | L | L | L | S | Some differences in baseline characteristics |
| Del Rosario | 2018 | L | L | L | L | L | Small sample size (n = 66) |
| Maddison | 2018 | L | S | L | L | S | High drop out rate (17% at 24 weeks) |
| Pandey | 2014 | S | L | S | S | H | Highly reliant on self-reported data |
| Pandey | 2014 | L | L | L | S | S | Highly reliant on self-reported data |
| Pfaefli Dale | 2015 | L | L | S | L | S | Highly reliant on self-reported data |
| Piotrowicz | 2010 | S | S | L | L | S | 20% drop out rate |
| Varnfield | 2014 | L | H | L | L | S | 40% drop out rate at six months |

H – high risk of bias; L- low risk of bias; S – some concerns
