# Supplementary material for: Mobile Phone Technologies in the Management of Ischemic Heart Disease, Heart Failure, and Hypertension: Systematic Review and Meta-Analysis
Source: JMIR Mhealth Uhealth. 2020 Jul 6;8(7):e16695. doi: 10.2196/16695 (PMC7381017; doi:10.2196/16695)
Supplement: Multimedia Appendix 4 [file mhealth_v8i7e16695_app4.docx]

| **Author** | **Year** | **n** | **Intervention** | **Follow Up Period** | **Effect on Compliance** |
| --- | --- | --- | --- | --- | --- |
| Fang | 2016 | 271 | SMS with or without a messaging app (Microletter) | 6 months | Odds ratio of non-compliance 0.07 (SMS+ML), 0.34 (SMS only), p < 0.001 |
| Khonsari | 2014 | 62 | SMS before every medication dose | 2 months | Rates of high adherence (MMAS-8): 65% vs. 13%  (p < 0.0001) |
| Park | 2015 | 90 | SMS with or without educational messages before every dose | 12 months | Higher percentage of correct doses for antiplatelets but not statins (88% and 87% vs. 72%, p <0.05) |
| Quilici | 2013 | 499 | Daily SMS | 1 month | Non adherence by patient interview: 2.8% vs. 7.2%,  p = 0.01; platelet function testing 5.2% vs. 11.2%, p = 0.01. |

SMS: short message service; app: application; BP: blood pressure
