# Supplementary material for: Mobile Phone Technologies in the Management of Ischemic Heart Disease, Heart Failure, and Hypertension: Systematic Review and Meta-Analysis
Source: JMIR Mhealth Uhealth. 2020 Jul 6;8(7):e16695. doi: 10.2196/16695 (PMC7381017; doi:10.2196/16695)
Supplement: Multimedia Appendix 5 [file mhealth_v8i7e16695_app5.docx]

| **Study** | **Year** | **n** | **Intervention** | **Follow up Period** | **Notable results favouring intervention** |
| --- | --- | --- | --- | --- | --- |
| Chen | 2019 | 767 | Daily educational SMS for 10 days, followed by weekly reminder SMS thereafter | 6 months | Reduced all-cause readmission rate (34% vs. 43%, p = 0.04).  Medication compliance 79% vs. 70% (p = 0.03) |
| Dendale | 2012 | 160 | Automated transmission of BP, HR and weight via mobile phone to a central server | 6 months | Decreased all-cause mortality (5% vs. 17%, p = 0.01) |
| Koehler | 2011 | 710 | Measurement of BP, weight and ECG and transmission via PDA | 26 months | Improved quality of life (SF-36 score; 54.3 vs. 49.9, P< 0.01) |
| Scherr | 2009 | 120 | Manual entry of BP and weight via mobile phone | 6 months | Functional status improvement by one NYHA class on average  Decreased length of stay when hospitalised  (6.5 vs. 10 days, p = 0.04). |
| Seto | 2012 | 100 | Remote BP and weight transmitted via Bluetooth and mobile phone | 6 months | Improved quality of life as measured by SCHFI score (73.3 vs. 65.5, p = 0.03) |
| Vuorinen | 2014 | 94 | Weekly transmission of weight and BP with symptom questionnaire | 6 months | No significant difference in any of the tested endpoints |

*BP: blood pressure; ECG: electrocardiogram; HR: heart rate; PDA: portable digital assistant; SMS: short message service*
