# Supplementary material for: Mobile Phone Technologies in the Management of Ischemic Heart Disease, Heart Failure, and Hypertension: Systematic Review and Meta-Analysis
Source: JMIR Mhealth Uhealth. 2020 Jul 6;8(7):e16695. doi: 10.2196/16695 (PMC7381017; doi:10.2196/16695)
Supplement: Multimedia Appendix 6 [file mhealth_v8i7e16695_app6.docx]

| **Study** | **Year** | **n** | **Intervention** | **Follow Up Period** | **Systolic BP reduction** |
| --- | --- | --- | --- | --- | --- |
| Bobrow | 2016 | 1372 | SMS information or interactive SMS | 12 months | 2.2 mmHg greater reduction than usual care (p = 0.04) |
| Kiselev | 2012 | 199 | SMS | 12 months | 23.7 mmHg vs. 6.9 mmHg (P < 0.05) |
| Logan | 2012 | 110 | Transmission of BP results with response from an automated system | 1 year | 7.1 mmHg greater reduction than the control group |
| Morawski | 2018 | 411 | Medisafe smartphone application (medication reminders) | 12 weeks | No significant difference |
| Morikawa | 2011 | 41 | Urine salt measurement and personalised email via mobile phone | 4 weeks | No significant difference |
| Varleta | 2017 | 314 | One SMS every 2 weeks | 6 months | No significant difference |

BP, blood pressure; SMS, short message system
