# Supplementary figures and images for: Mobile Phone Technologies in the Management of Ischemic Heart Disease, Heart Failure, and Hypertension: Systematic Review and Meta-Analysis
Source: JMIR Mhealth Uhealth. 2020 Jul 6;8(7):e16695. doi: 10.2196/16695 (PMC7381017; doi:10.2196/16695)

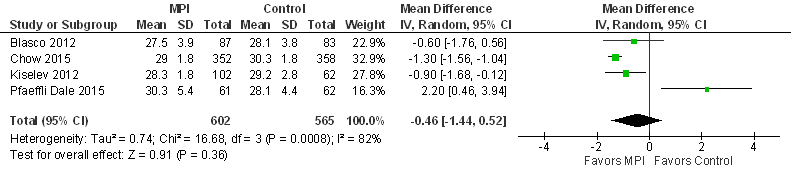

Supplement: Multimedia Appendix 7 [file mhealth_v8i7e16695_app7.png]
